# A novel medical image segmentation approach by using multi-branch segmentation network based on local and global information synchronous learning

**DOI:** 10.1038/s41598-023-33357-y

**Published:** 2023-04-25

**Authors:** Shangzhu Jin, Sheng Yu, Jun Peng, Hongyi Wang, Yan Zhao

**Affiliations:** 1 Information Office, Chongqing University of Science and Technology, Chongqing, 401331 China; 2 College of Intelligent Technology and Engineering, Chongqing University of Science and Technology, Chongqing, 401331 China; 3 College of Mathematics, Physics and Data Science, Chongqing University of Science and Technology, Chongqing, 401331 China

**Keywords:** Medical research, Computer science

## Abstract

In recent years, there have been several solutions to medical image segmentation, such as U-shaped structure, transformer-based network, and multi-scale feature learning method. However, their network parameters and real-time performance are often neglected and cannot segment boundary regions well. The main reason is that such networks have deep encoders, a large number of channels, and excessive attention to local information rather than global information, which is crucial to the accuracy of image segmentation. Therefore, we propose a novel multi-branch medical image segmentation network MBSNet. We first design two branches using a parallel residual mixer (PRM) module and dilate convolution block to capture the local and global information of the image. At the same time, a SE-Block and a new spatial attention module enhance the output features. Considering the different output features of the two branches, we adopt a cross-fusion method to effectively combine and complement the features between different layers. MBSNet was tested on five datasets ISIC2018, Kvasir, BUSI, COVID-19, and LGG. The combined results show that MBSNet is lighter, faster, and more accurate. Specifically, for a $320 \times 320$ input, MBSNet’s FLOPs is 10.68*G*, with an F1-Score of $85.29\%$ on the Kvasir test dataset, well above $78.73\%$ for UNet++ with FLOPs of 216.55*G*. We also use the multi-criteria decision making method TOPSIS based on F1-Score, IOU and Geometric-Mean (G-mean) for overall analysis. The proposed MBSNet model performs better than other competitive methods. Code is available at https://github.com/YuLionel/MBSNet.

## Introduction

Medical images such as ultrasound (US) and magnetic resonance imaging (MRI) are widely used in clinical diagnosis now. Automatic segmentation of medical images can provide pathological analysis for doctors and play a major part in modern and intelligent medical care. Since deep convolutional neural networks can learn and extract useful information from data, they are currently the mainstream in the application of medical image segmentation. Compared with traditional methods, deep learning networks can learn and use feature information more autonomously and efficiently.

Recently, medical image segmentation research has shown that there are mainly three solutions: (1) U-shape method. The U-shape method can reduce the loss caused by spatial changes to a certain extent through deep mapping and skip connection structure and can learn deep semantic information. UNet^1^ is one of the most influential networks. Many medical image segmentation networks draw on the idea of combining the structure of UNet encoder–decoder with skip connections, such as UNet++^2^, R2U-Net^3^, UNeXt^4^, CA-Net^5^, AttU-Net^6^ and so on. From the analysis in^7^, it is concluded that although these network architectures can make up for the loss of low-level features, it is inevitable to lose many spatial details through deep sacrifice resolution downsampling learning, resulting in reduced prediction accuracy. (2) Transformer-based method. Since the Dosovitskiy team^8^ proposed the vision transformer (ViT), it has caused a sensation in the field of visual vision. Its ability to model distant relationships tends to overtake deep convolutional neural networks (CNNs) in classification and segmentation tasks. The global information of the Transformer^9^ structure on the image brings new ideas to many scholars. For example, Swin transformer^10^ introduces the ViT structure into the sliding window mechanism and uses a hierarchical design to expand the receptive field and increase the locality. Transunet^11^ combines UNet and transformer to recover local information extraction. These works have good performance, but their real-time performance and computational complexity are poor due to many parameters. (3) Multi-scale feature learning. Most of these networks use image pyramids to collect multi-scale information, such as^12^ extended pooling, which enhances the object information in different features and merges diverse scale features. Yang et al.^13^ Using multi-scale images while adding attention to training achieves excellent performance. Chen et al.^14^ proposed an ASPP module to capture the global context of an image, using inflated convolutions of different coefficients to extend the learning feature scale. However, most of these proposed networks have the same problem as they only perform multi-scale extraction on the last stage of the Encoder process, while many low-level features have been lost at this stage.

With the application of medical imaging technology in medical diagnosis and treatment, the requirements for a segmentation network is not only limited to its accuracy but also its lightness and speed. Therefore, we propose a multi-branch synchronous learning segmentation network based on local and global information. The local feature extraction branch (L) includes a 5-layer convolution block, which can effectively learn complex semantic information. The number of convolution layers of the global feature extraction branch (G) reduces to 3 compared with the traditional encoder, which avoids the loss of a large number of spatial details due to the massive compression of spatial dimensions caused by too deep convolution. In order to guide the two branches to learn local information and global information better, branch L adds a parallel residual mixer (PRM) each time after using ordinary convolution, and branch G embeds a dilated convolution with multi-grid parameters to expand the receptive field in each convolution block. The research results in^15^ show that local information can help to obtain low-level features, so we also fuse the information in branch L of the same level as in branch G of the Encoder stage. In addition, we use a feature cross-fusion block (FCFB) in the Decoder phase to better complement the two features.

What we have done can be illustrated as follows:

- We propose MBSNet, a new multi-branch segmentation network that can efficiently utilize local and global information in images.
- We design a new spatial attention module and a feature cross-fusion method that can effectively enhance feature learning.
- This research achieved excellent results at ISIC2018^16^, Kvasir^17^, Breast UltraSound Images (BUSI)^18^, COVID-19^19^, and LGG^20^ with F1-Scores of $87.76\%$, $85.29\%$, $72.81\%$, $76.25\%$, and $69.57\%$, respectively.

## Related work

### U-shape method

Upsampling directly from low resolution leads to the loss of a large amount of spatial information, and the results are relatively rough, like FCN^21^. Recently, many methods have adopted the U-shape structure. UNet^1^, a landmark work, combines multi-level feature upsampling with skip connection to reduce the loss of spatial information. Inspired by ResNet, Chaurasia et al.^22^ proposed Linknet, which adds a residual structure to the original UNet and retains lost information from different layers in the coding part. Zhou et al.^2^ improved the UNet architecture by adding multiple skip connections to aggregate diverse scale features. Zhang et al.^23^ proposed DENS-INception U-net, which uses dense connections in the network and has a good performance in medical image segmentation. Alom et al.^3^ proposed R2U-Net using cyclic residual convolutional layers to learn more representative features. The role of the U-Shape structure in medical image segmentation tasks has been strongly confirmed. However, the model of this structure often loses the spatial information in the image, which is not conducive to restoring the edge details of the target area.

**Figure 1:**
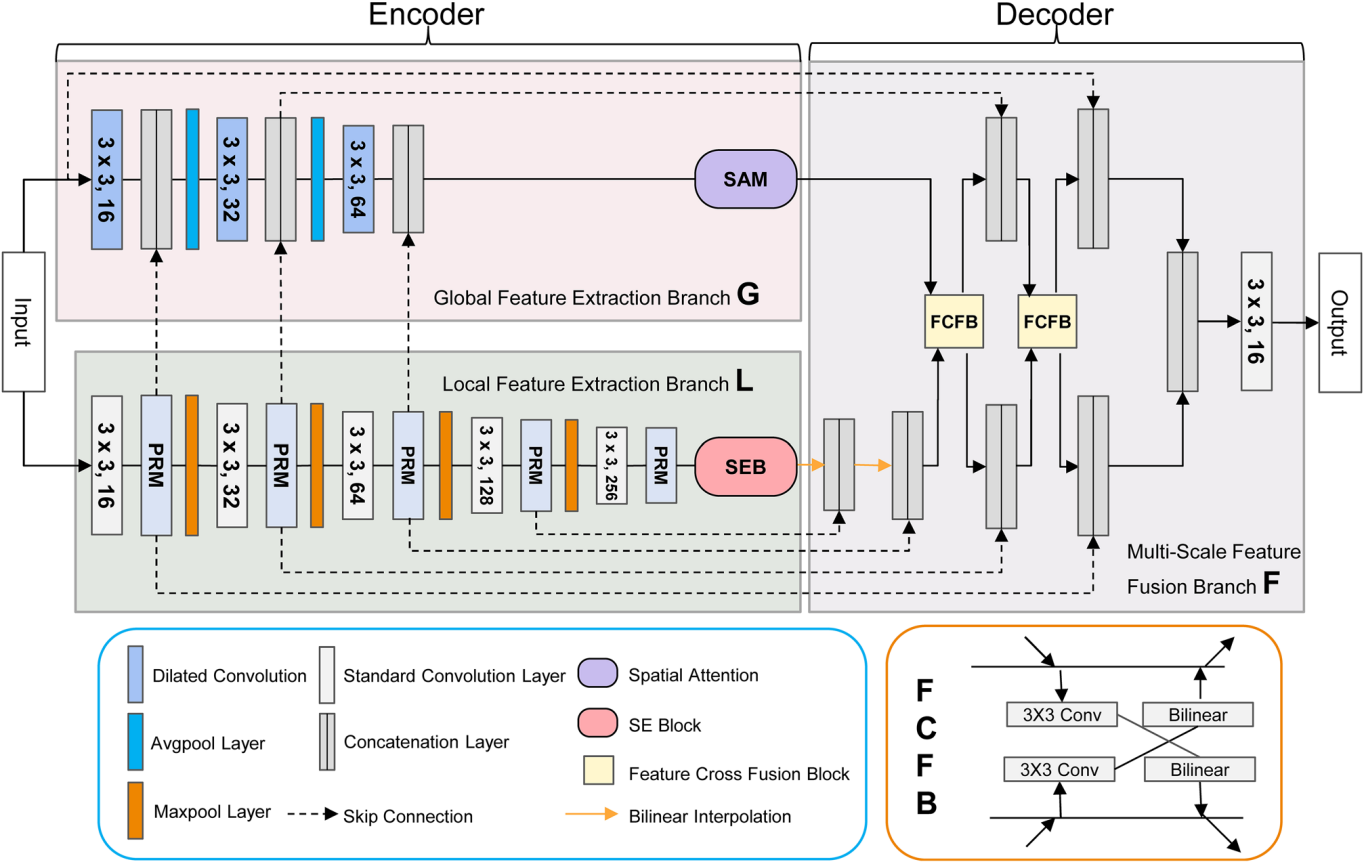
Overview of the proposed MBSNet architecture. The upper part of the Encoder phase is branch G, the lower part is branch L, and the Decoder phase is branch F. We also place the structural details of the FCFB in the lower right corner. In the FCFB, the features of the two branches are cross-added to different branches to learn complementarity.

### Multi-branch learning

Multi-branch learning aims to learn different features through multiple branches to refine the prediction results and improve the segmentation performance. KiU-Net^7^, an over-complete convolution structure, maps input features to higher dimensions, and extracts fine details of boundaries and small structures, which is contrary to traditional operation mapping. BASNet^24^consists of a dense encoder-decoder network and a light encoder–decoder network for predicting and refining segmentation probabilities, respectively. OCTA-Net^25^ applies two modules to generate preliminary confidence maps and further optimize the contour of the segmented object. MODNet^26^ decomposes the matting task design into three sub-tasks and optimizes them simultaneously through specific constraints to achieve real-time fine-grained portrait matting. Most of these networks learn to refine and get the final results through different sub-tasks and show good progress in accuracy. Although these methods learn the target region features hierarchically through the idea of multiple branches, the interpretability of their internal working principles is not sufficient. For example, KiU-Net only attempts to limit the increasing receptive field during convolution. BASNet further refines the prediction network through a deeper fine network.

### Attention mechanism

The attention mechanism can guide the convolutional network to learn correct knowledge and suppress invalid regions. BiSeNet^27^ applies the attention refinement module to the extracted features with low computational cost and improved accuracy. Qin et al.^28^ proposed autofocus convolutional layer, which fuses feature maps of different sizes in the middle layer by weighting to extract multi-scale information. MODNet^26^ adds SE-Block^29^ to the encoding process and re-weights the feature map. CA-Net^5^ uses multiple attention and is fully integrated to improve the accuracy of network perception of target location and size. However, although many existing image segmentation networks have applied attention mechanisms to improve their performance, they only serve one or two datasets. In addition, such networks often require more computing resources and training time.

### Global and local information

In recent years, the application of high-frequency and low-frequency features in images have become the main research direction, and high-frequency and low-frequency features can also be called local information and global information. These algorithms^30–32^ use different methods to separate global and local features. The difference between local and global components is that global information contains the global shape and structure of the image, while local information pays more attention to the texture change of the image. Dosovitskiy^8^ proposed ViT, through which self-attention can excellently capture global dependencies and obtain global information. Inception Transformer^31^ adopts the structure of ViT and proposes a new hybrid architecture (Inception mixer), which flexibly fuses global and local information. However, it directly outputs the upper layer features and combines them with explicit fusion modules, lacking different frequency feature learning. Bai et al.^33^ proposed MF$^2$CNet, which improves the fusion of multi-frequency features to improve network performance. Based on the above research, it can be seen that effective learning of global and local information in the data during the feature extraction process can indeed obtain more accurate segmentation results, but the number of parameters and computational complexity of such models are much larger than common segmentation models.

MBSNet draws on the concept of high and low-frequency information capture and combination, but the difference is that we use dilated convolution and average pooling operations to obtain global features, and only supplement local information with global information in the encoder stage, while local information does not combine global information. This is done to learn more about pure semantic information and reduce network complexity.

## Proposed MSBNet

Some existing approaches^12,34,35^ for segmentation tasks obtain multi-scale feature refinement results through deep convolution and a skip connection network structure. In addition, some methods^5,27,36^ use attention modules to emphasize the response of foreground regions and calibration channels to make the network more adaptable. These methods have proved that multi-scale information and attention mechanism are effective for segmentation tasks.

In this section, we elaborate on the details of the proposed MBSNet. As shown in Fig. 1, MBSNet consists of three stages: local feature extraction branch (L), global feature extraction branch (G), and multi-scale feature fusion branch (F). Inspired by the structure of Inception Mixer^31^, in the process of extracting feature information from a deep convolutional neural network^37^, low-level features are generalized to supplement local information, while high-level features are complex and need to supplement global information. Therefore, we propose a depth-aware global and local information network that performs better on pixel-level prediction tasks.

First, branches L and G learn and output the local and global information of the image simultaneously. Secondly, branch G timely complements the local features of the corresponding level of L. Finally, branch F at the end of MBSNet fuses the features and obtains the segmentation results.

**Figure 2:**
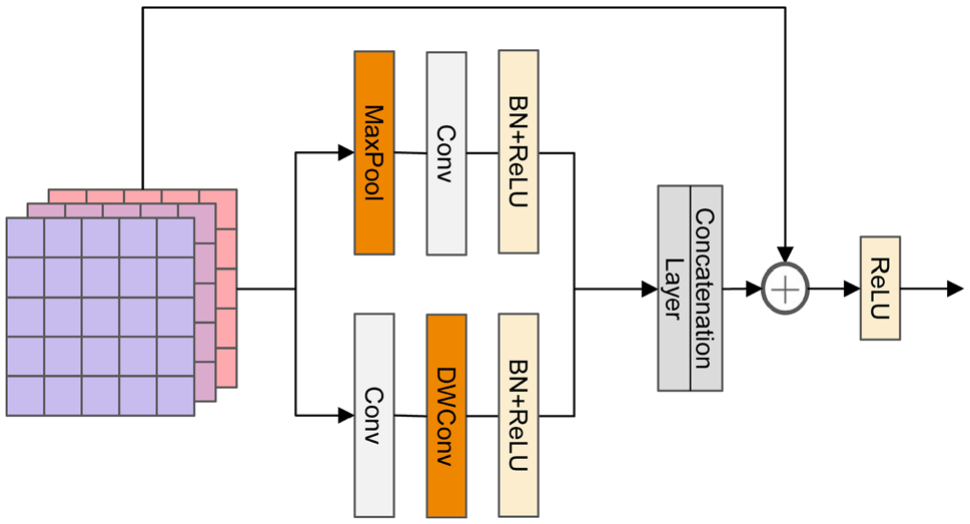
Structure of PRM. It includes two parts, max pooling operation and a depth wise convolutional layer. The role of PRM is to enhance the capture of local information, and the residual structure can retain more semantic information.

### Local feature extraction branch

Branch L is used to capture the local information of the input image ${X}\in \mathbb {R}^{H \times W \times C}$, where $C=3$. Similar to the encoder part of many existing convolutional networks, it is also a five-layer convolutional block. However, different from the standard UNet, each Block is reduced from 2 convolutions to 1 convolution, and the number of channels is reduced by 1/4, respectively 16, 32, 64, 128, 256. After the above changes, the proposed module can not only retain effective semantic information but also reduce the number of parameters and save computing resources and time. We add a parallel residual mixer (PRM) after each convolutional layer to further extract local information from the convoluted features. In addition, maximum pooling is used to reduce the feature dimension. We note that branch L can mine different channel characteristics, so we use SE-Block^29^ to weigh each channel to guide the learning of correct knowledge to improve network learning performance.

As shown in Fig. 2, the structure of PRM consists of two parts: a max pooling operation and a depth-wise convolutional layer (DWConv). The max pooling can select features with higher classification recognition, so it can retain more local information and correct the numerical offset caused by the error of convolution parameters before. The other part uses DWConv for two reasons: (1) DWConv helps to perceive the details of semantic information. (2) It has fewer convolution operation parameters. In DWConv, we use layer normalization (LN) to normalize the channel layer of a single sample and use the Gaussian error linear unit activation function (GELU) as the activation layer between convolutional layers. GELU is used in recently studied networks such as ViT^8^ and MLP-Mixer^38^ and is more efficient than Relu. PRM can be denoted as:

1 $$\begin{aligned} Y_{\text{ prm } }=f_{3 \times 3}\left\{ f_{1 \times 1}\left( F_{m p}(X)\right) \bigoplus F_{d c}\left( f_{1 \times 1}(X)\right) \right\} +X. \end{aligned}$$

where $f_{1 \times 1}(\cdot )$ and $f_{3 \times 3}(\cdot )$ are standard 1 × 1 and 3 × 3 convolution layers followed by batch normalization^39^ and ReLU activation, $F_{m p}(\cdot )$ is a max pooling operator, $F_{d c}(\cdot )$ denotes depth-wise convolution and $\bigoplus$ indicates concatenation, and $+$ means element-wise addition operation.

**Figure 3:**
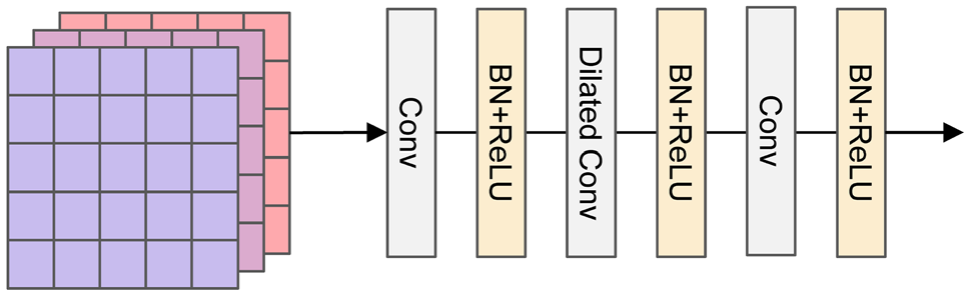
Structure of dilated convolution block. The block extends the field of view through dilated convolutions, providing the ability to perceive global information.

### Global feature extraction branch

There are four main differences between branch G and branch L: (1) Multiple dilated convolution blocks are embedded in the branch G. As shown in Fig. 3, each dilated convolution block has two standard convolution layers and a dilated convolution layer between the two convolution layers. Motivated by^14,40,41^, the dilated convolution uses different sizes of dilated rates. Set the hyperparameters r = 2, Multi_Grid = (1, 2, 4), then dilated rates = $2\times (1,2,4) = (2,4,8).$ (2) Branch G has only three convolution blocks. For fine-grained pixel-level segmentation tasks, the size of the feature mapping connecting the encoder and the decoder is usually small, which causes information loss, so we only downsample twice. (3) The pooling operation between each convolution block uses average-pooling because compared with max-pooling, average-pooling retains more complete data, that is, it can retain the global information in the feature. (4) The local information of the corresponding level in L is also fused in the downsampling process. Formally, we assume that *X* is an input feature map and $f(\cdot )$ is a ${1 \times 1}$ convolutional layer, and $F_{g}(\cdot )$ is an inflated convolution, both containing BN^39^ and ReLU, the formula is as follows:

2 $$\begin{aligned} Y=f\left( F_{g}(f(X))\right) . \end{aligned}$$

**Figure 4:**
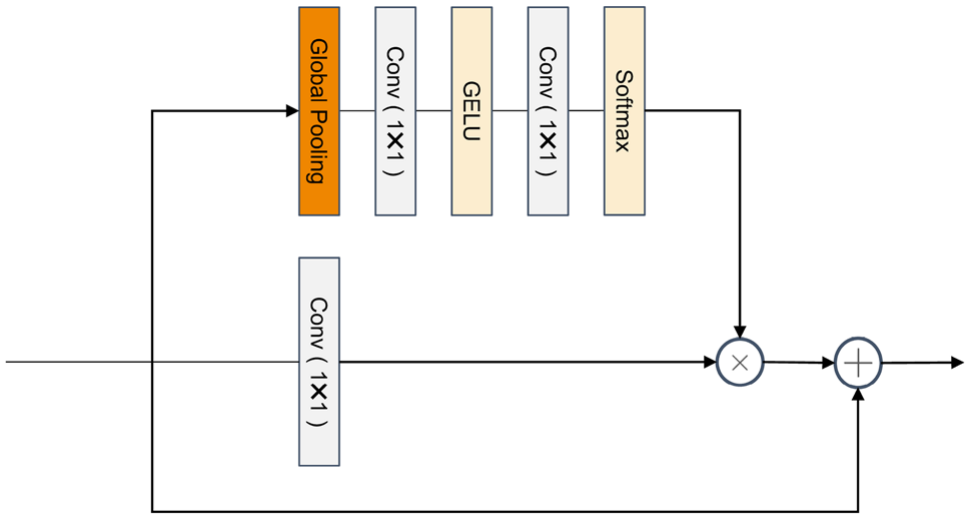
The details of spatial attention module.

As shown in Fig. 4, we propose a spatial attention module (SAM) to enhance the spatial information of the output of the features by branch G to integrate the global contextual information. Motivated by^8,42,43^, SAM converts the input features into Q, V, K features. Matrix multiplication is performed on Q and V, and K is added after reshaping. This design can enhance the perception of branch G to spatial dimension information and better capture spatial dependencies. Compared with self-attention, the computational complexity is reduced and thus have good results.

Formally, $F_{g l}(\cdot )$ is an adaptive average pooling operator, $F_{s m}(\cdot )$ denotes softmax operator, and $F_{g e}(\cdot )$ is the GELU operator, and $\otimes$ is the matrix dot product operation.

3 $$\begin{aligned} Y_{\text{ sam } }=\left\{ f(X) \otimes F_{s m}\left[ f\left( F_{g e}\left( f\left( F_{g l}(X)\right) \right) \right) \right] \right\} +X . \end{aligned}$$

### Multi-scale feature fusion branch

Branch F is used to fuse the global and local information output by branches L and G. Since the mapping depth of branch L is deeper than that of branch G, the 4th and 5th layers of L are first sampled by bilinear interpolation and supplemented by skip connections. To better fuse large-scale information, we design a Feature Cross Fusion Block (FCFB) to further complement the different features extracted by the two branches.

The FCFB structure is shown in Fig. 1. The features from the $\dot{1}$ layer in branches G and L are represented as $Y_{G}^{i}$ and $Y_{L}^{i}$, which are then mapped using an upsampled convolution block containing a standard convolution layer and a bilinear interpolation upsampled layer, respectively. Finally, the output $\widehat{Y_{G}^{i}}$ and $\widehat{Y}_{L}^{i}$ are used for another branch by crossing. The advantage of the FCFB is that it can complement the features with a small amount of calculation, which is more conducive to training.

## Experiments

### Implementation and evaluation methods

All experiments used the Pytorch framework and were implemented on the RTX 3060 GPU. The batch size was 4, and each image was resized to 320 $\times$ 320 and normalized by mean and standard deviation. We used an Adam as the network optimizer and set the initial learning rate to 0.001. Additionally, we used a cosine annealing learning rate scheduler with a minimum learning rate as high as 0.00001. Horizontal flipping, vertical flipping, and random cropping were used to augment the data. We used a joint loss function of cross entropy (BCE) and dice loss for training and retaining the best-performing model on the validation set across all epochs. Finally, the prediction $\hat{Y}$ is trained by our target *Y* with the following loss $\mathcal {L}$:

4 $$\begin{aligned} \mathcal {L}=0.5 \mathcal {L}_{\text{ bce } }(\hat{Y}, Y)+\mathcal {L}_{\text{ dice } }(\hat{Y}, Y) . \end{aligned}$$

We selected several common evaluation indicators to evaluate our network segmentation performance, including F1-Score, IOU, and G-mean score^44^. In addition, we compared the number of parameters and FLOPs with baselines. The formula of IOU is:

5 $$\begin{aligned} I O U=\frac{\mathcal {R}_{a} \cap \mathcal {R}_{b}}{\mathcal {R}_{a} \cup \mathcal {R}_{b}} . \end{aligned}$$

where $\mathcal {R}_{a}$ and $\mathcal {R}_{b}$ represent the network prediction results and ground truth, respectively. In order to comprehensively evaluate the quality of the network, F1-Score, G-mean score, which is defined as:

6 $$\begin{aligned} F 1=\frac{2 \times \text{ Precision } \times \text{ Recall } }{ \text{ Precision } + \text{ Recall } }. \end{aligned}$$

7 $$\begin{aligned} G-\text {mean}=\sqrt{ \text{ Sensitivity } \times \text{ Specificity } }. \end{aligned}$$

where Precision = $TP/(TP+FP)$, Recall = $TP/(TP+FN)$, Specificity = $TN/(TN+FP)$. *TN* is true negative, *FP* is false positive, *TP* is true positive, *FN* is false negative.

**Figure 5:**
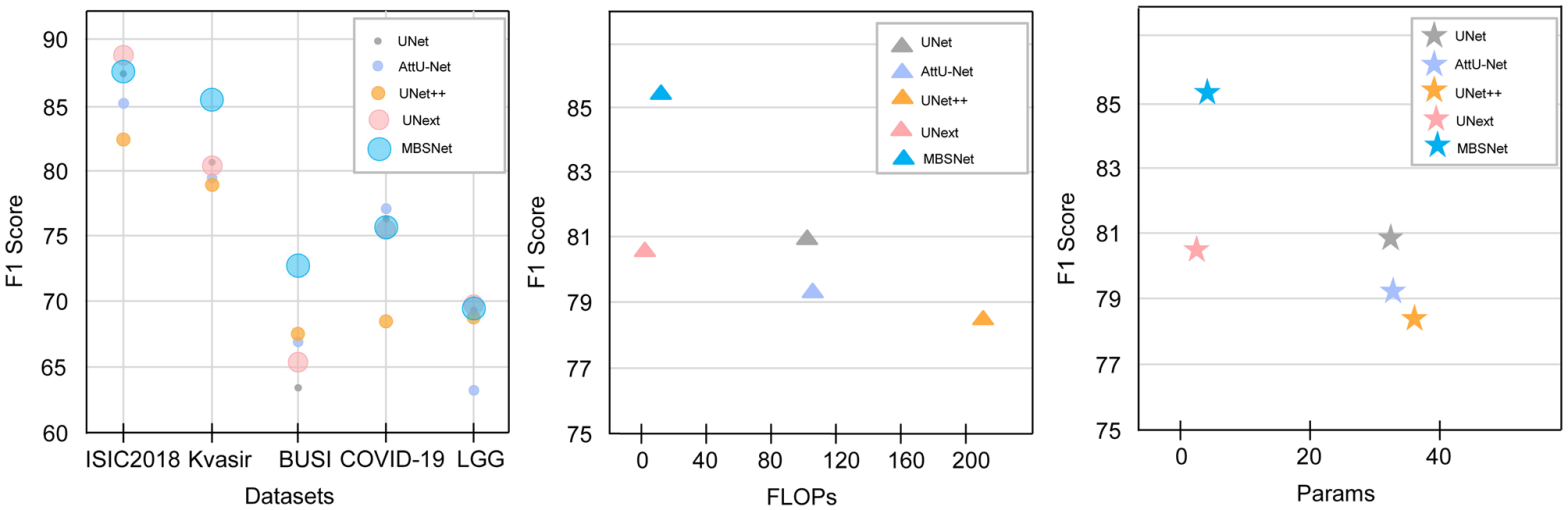
The first image shows the F1-Score of each network on five datasets. The Y axis of the second and third images is the F1-Score of each network on the Kvasir dataset, and the X axis corresponds to FLOPs and parameters, respectively. MBSNet is the best network in terms of overall comparison.

### Segmentation of multiple datasets

#### ISIC2018 dataset

Medical boundary segmentation can assist and improve the effectiveness and accuracy of clinical diagnosis. We used the ISIC2018 dataset^16^ released by the International Skin Imaging Collaboration (ISIS) to evaluate MBSNet, which contains 2594 color dermoscopy images for training, 100 test images, and the corresponding ground truth. We randomly divided the dataset into 2205, 389 and 100 for training, validation and testing, respectively.

We compared MBSNet with multiple networks, such as UNet^1^, UNet++^2^, AttU-Net^6^, and UNeXt^4^. All training hyperparameter settings are described as in subsection 3.1. MBSNet obtained higher scores in IOU score than most comparison networks, obtaining 80.17, indicating that MBSNet has a good segmentation performance on skin lesion segmentation. In addition, the number of parameters of MBSNet is only 3.98 million, which is much smaller than that of UNet (34.53 M).

**Figure 6:**
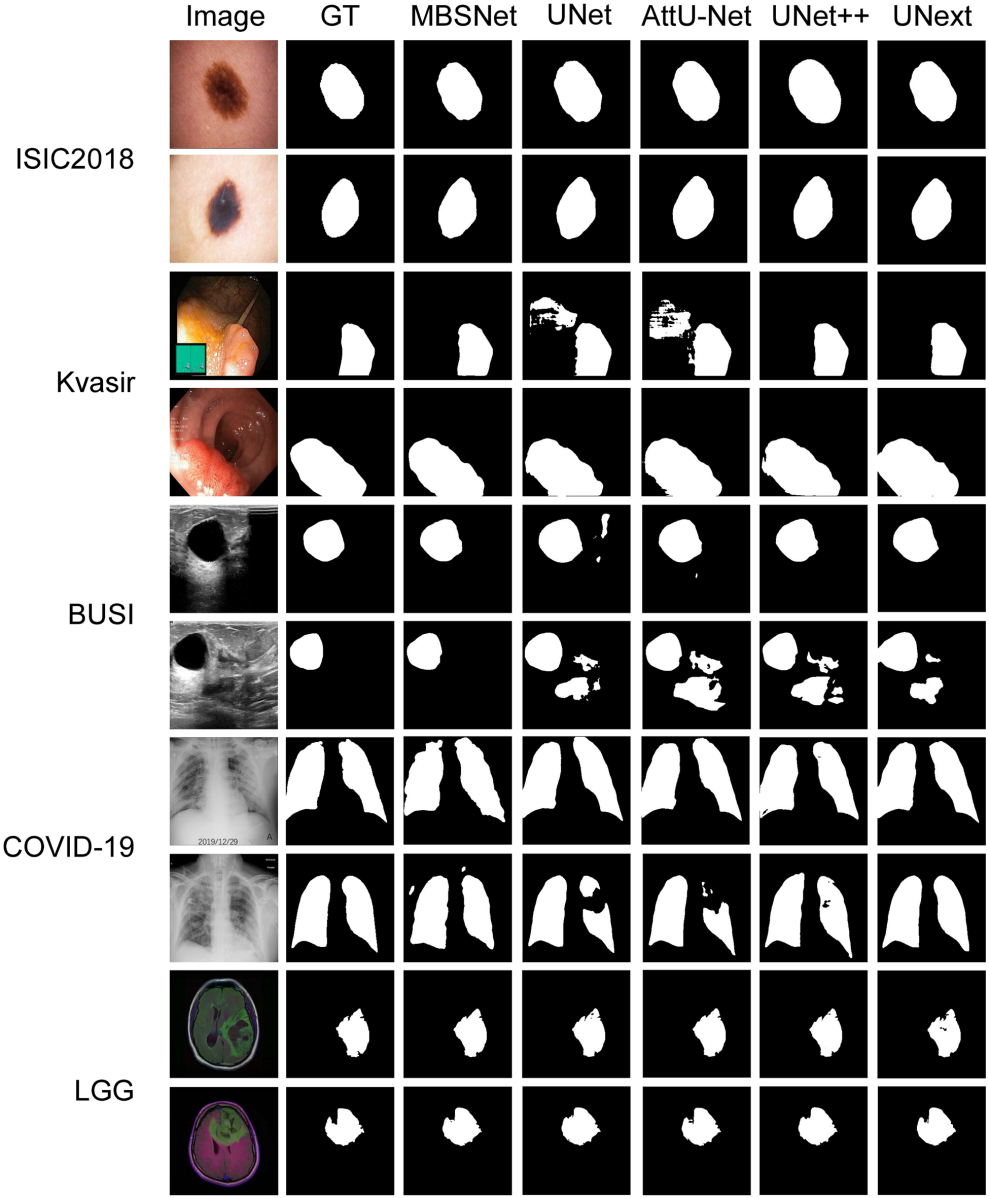
MBSNet is compared with five other networks on ISIC2018 dataset, Kvasir dataset, BUSI dataset, COVID-19 dataset and LGG dataset. Obviously, MBSNet can perceive the shape structure of the target and effectively suppress the misleading of error information to obtain accurate prediction results.

To get a more intuitive understanding of the performance advantages of the networks, we draw Table 1 to quantitatively compare different networks. The size of each model parameter and the computational complexity are shown in Table 2. Figure 5 clearly shows the corresponding quantitative results of MBSNet and comparison networks on the ISIC2018 dataset. Figure 6 shows the qualitative comparison results with other baselines on ISIC2018, and it can be seen that MBSNet has good prediction results. In the quantitative analysis, we also used the TOPSIS^45–48^ algorithm to obtain the final model score for the three indicators of F1, IOU, and G-mean to find the best model. Although MBSNet ’s score is not the highest, it is only slightly lower.

**Table 1:** Quantitative comparison of MBSNet with other models on ISIC2018 dataset, Kvasir dataset, BUSI dataset, COVID-19 dataset, and LGG dataset.

		F1	IOU	G-mean	TOPSIS
ISIC2018	UNet^1^	87.21	79.15	92.54	0.23829325
	AttU-Net^6^	85.69	76.95	92.27	0.14954534
	UNet++^2^	83.04	73.95	90.08	0
	UNeXt^4^	**88.71**	**81.22**	**93.7**	**0.33225513**
	MBSNet	87.76	80.17	92.99	0.27990629
Kvasir	UNet^1^	80.9	71.99	89.56	0.18503661
	AttU-Net^6^	79.29	69.73	89.27	0.04927065
	UNet++^2^	78.73	69.26	88.49	0
	UNeXt^4^	80.79	72.5	88.6	0.19576564
	MBSNet	**85.29**	**77.66**	**92.1**	**0.5699271**
BUSI	UNet^1^	63.68	53.12	79.92	0.02332205
	AttU-Net^6^	66.93	56.67	**82.51**	0.18599036
	UNet++^2^	67.7	57.4	80.93	0.210357
	UNeXt^4^	65.94	55.22	79	0.10551958
	MBSNet	**72.81**	**63.21**	82.16	**0.47481101**
COVID-19	UNet^1^	76.78	65.93	89.06	0.26129017
	AttU-Net^6^	**77.01**	**66.06**	89.54	**0.27014396**
	UNet++^2^	68.61	56.37	84.88	0
	UNeXt^4^	75.69	64.91	87.26	0.2220694
	MBSNet	76.25	65.13	**89.59**	0.24649646
LGG	UNet^1^	69.03	59.55	79.06	0.25013003
	AttU-Net^6^	63.26	54.47	72.4	0
	UNet++^2^	68.85	58.37	78.97	0.22191493
	UNeXt^4^	**69.71**	59.59	**79.84**	0.26303922
	MBSNet	69.57	**60.23**	79.1	**0.26491581**

Significant values are in [bold].

**Table 2:** Comparison of computational complexity and number of parameters between MBSNet and each comparison model.

Methods	Year	FLOPs (G)	Params (M)
UNet^1^	2015	102.56	34.53
AttU-Net^6^	2018	104.28	34.88
UNet++^2^	2018	216.55	36.63
UNeXt^4^	2022	**0.87**	**1.47**
MBSNet	2023	10.68	3.98

Significant values are in [bold].

**Table 3:** Best model ranking.

	ISIC2018	Kvasir	BUSI	COVID-19	LGG	Average
UNet^1^	3	3	5	2	3	3.2
AttU-Net^6^	4	4	3	**1**	5	3.4
UNet++^2^	5	5	2	5	4	4.2
UNeXt^4^	**1**	2	4	4	2	2.6
MBSNet	2	**1**	**1**	3	**1**	**1.6**

Significant values are in [bold].

MBSNet and other comparison models are ranked by TOPSIS scores on multiple datasets.

#### Kvasir dataset

Related studies have shown that in colonoscopy, the missed diagnosis rate of polyps is 14–30 %, which has a huge hidden danger to human health. The main motivation of the Kvasir dataset^17^ is to automatically detect polyps in the human gastrointestinal, which plays an important role in the treatment of colorectal cancer. Kvasir contains gastrointestinal polyp images and ground truth, with an image size ranging from 332 $\times$ 487 to 1920 $\times$ 1072 pixels, a total of 1000, and manually annotated by clinical experts. We randomly divided the dataset into training set, validation set and test set, and get 700, 100 and 200, respectively.

In Fig. 6, it shows the segmentation results of several networks on the Kvasir dataset. We observed that the gastrointestinal tract is close to the color of the polyp, which makes networks like UNet mistakenly believe that the region is also a polyp. This has a great negative impact on prediction results.

The quantitative results are presented in Table 1, and the comparison of F1-Scores and the number of parameters is shown in Fig. 5. It can be seen that MBSNet combines the characteristics of local information and global information, and achieves good comprehensive performance. Note that the TOPSIS score of MBSNet on the Kvasir dataset is much higher than other comparison models, which proves the superiority of MBSNet network.

#### Breast ultrasound images dataset

Breast ultrasound images (BUSI) dataset^18^ are for breast cancer and include normal, benign, and malignant breast ultrasound images and real-world conditions in women aged 25–75. We only selected a total of 630 benign and malignant images. We randomly divided the dataset into training set, validation set and test set, and get 441, 63 and 126, respectively.

From Table 1, we can see the quantitative comparison results of MBSNet and other networks trained in a consistent environment on the BUSI dataset. Figure 6 shows the prediction results of MBSNet and other methods on the BUSI dataset. It can be seen that UNet and its variants have a vague perception of the boundary of the target area. MBSNet is hardly affected by noise and other factors in ultrasonic images, and the segmentation results are closer to ground truth. We also use TOPSIS to score the model, and the result is that MBSNet has the best effect on the ultrasonic mode dataset.

#### COVID-19 dataset

In 2019, Corona Virus Disease 2019 (COVID-19) caused a global infectious infection^19^. However, common virus analysis methods are expensive and time-consuming, and not suitable for dealing with a large number of patients. Therefore, a rapid and effective diagnostic method is imminent. Considering the impact of COVID-19 on lung tissue, chest X-ray (CXR) can be used as a technical solution for screening and detecting COVID-19. The dataset contains 2913 images of COVID-19 CXRs and corresponding masks. It is a subset of the COVID-QU-Ex dataset, compiled by researchers at the University of Qatar. Among them, 1864 samples were used for training, 466 for verification, and 583 for testing.

The segmentation prediction results are shown in Fig. 6, and the quantitative comparison results with the comparison method are shown in Table 1. Among them, MBSNet has achieved good results in F1, IOU and G-mean indicators, which are 76.25%, 65.13%, and 89.59%, respectively. In summary, the method in this research can also have better performance in the dataset of CT mode.

#### LGG segmentation dataset

The LGG segmentation dataset^20^ is from The Cancer Imaging Archive (TCIA), including brain MR images and corresponding segmentation masks of 110 patients. The size of MRI slice images of each patient was 256 $\times$ 256, but the number varied greatly. For the effectiveness of model training, images without lesions were deleted from LGG. Finally, the remaining dataset contains 988 for training, 110 for validation and 275 for testing.

Figure 6 shows the segmentation prediction of LGG dataset by MBSNet, UNet, UNet++ and other networks. It can be seen that MBSNet can segment the lesion area more completely than other networks. In addition, Table 1 shows the quantitative results of each network, and MBSNet ranks first in IOU and TOPSIS indicators. This shows that MBSNet can obtain the feature information in the graph and restore it more effectively.

#### Results analysis

We compared MBSNet with existing methods on five different datasets. The results show that MBSNet has achieved good results on multiple data sets, and the segmentation effect is obvious. In addition, the computational complexity and the number of parameters are greatly reduced. However, on the COVID-19 dataset, the effect of MBSNet is not significant. The TOPSIS score ranking of different models on five datasets is shown in Table 3. The average ranking of MBSNet on all rankings of each dataset is the first, which proves that MBSNet has good results in the datasets of dermoscopy, gastroscopy, colonoscopy, ultrasound (US), computed tomography (CT), and nuclear magnetic resonance (MRI) under five different modes, and is a general medical image segmentation method.

**Figure 7:**
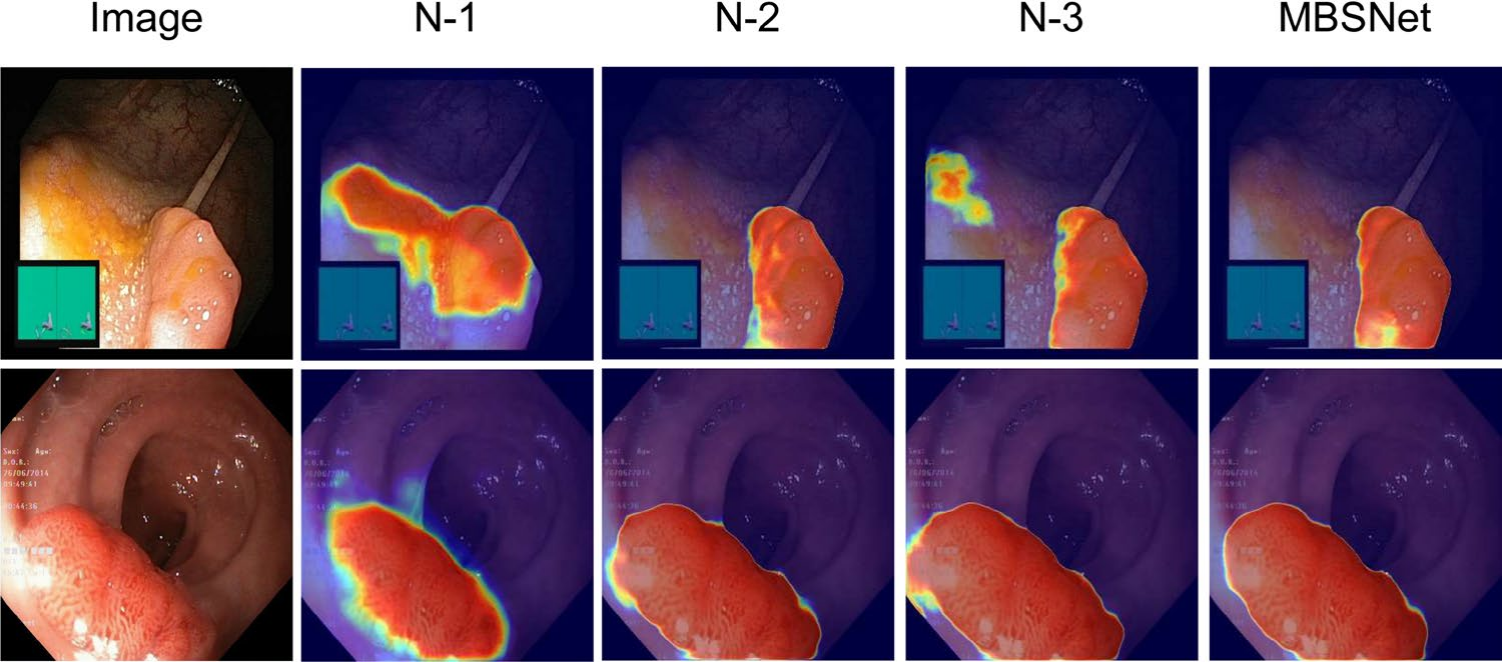
Activation diagram of MBSNet with each component added. Warmer the color, greater the proportion of attention in the area. N − 1 refers to only with a dilated convolution component, N − 2 represents an addition of PRM to the N − 1, N − 3 adds one more SE-Block than the N − 2.

### Ablation study

In this section, we conducted an ablation study to better understand the effectiveness of each component and branch in MBSNet. This ablation experiment was performed on the Kvasir dataset^17^. All training used the same settings as described in “Implementation and evaluation methods” and we trained a total of 100 epochs.

**Table 4:** Ablation analysis of the individual components of the proposed architecture on the Kvasir dataset.

Dilated Conv	PRM	SEB	SAB	FLOPs(G)	Params(M)	F1	IOU
$\checkmark$				6.92	1.38	78.38	68.87
$\checkmark$	$\checkmark$			10.65	3.83	80.77	72.26
$\checkmark$	$\checkmark$	$\checkmark$		10.65	3.97	84.67	76.91
$\checkmark$	$\checkmark$	$\checkmark$	$\checkmark$	10.68	3.98	85.29	77.66

Each of our components contributes differently to the results.

#### Ablation for each module in MBSNet

In order to improve the perception ability of the whole network, several components are added, and Table 4 shows the results of the quantitative comparison. Among them, after adding the PRM module, the F1-Score and IOU have a big leap, $80.77\%$ and $72.26\%$ respectively, which shows the effectiveness of PRM. After that, SE-Block and SAB are added to further improve the accuracy rate, and the computational complexity do not increase much.

Figure 7 visualizes the activation maps of MBSNet with each component added to the Kvasir dataset^17^. It can be seen that the network’s learning of polyps is biased when only dilated convolution is used, and the positioning object can be obtained more completely after PRM is added. The addition of SE-Block and SAB proves the effectiveness of guided training for feature learning. The joint learning of these key parts can indeed predict polyps completely.

**Figure 8:**
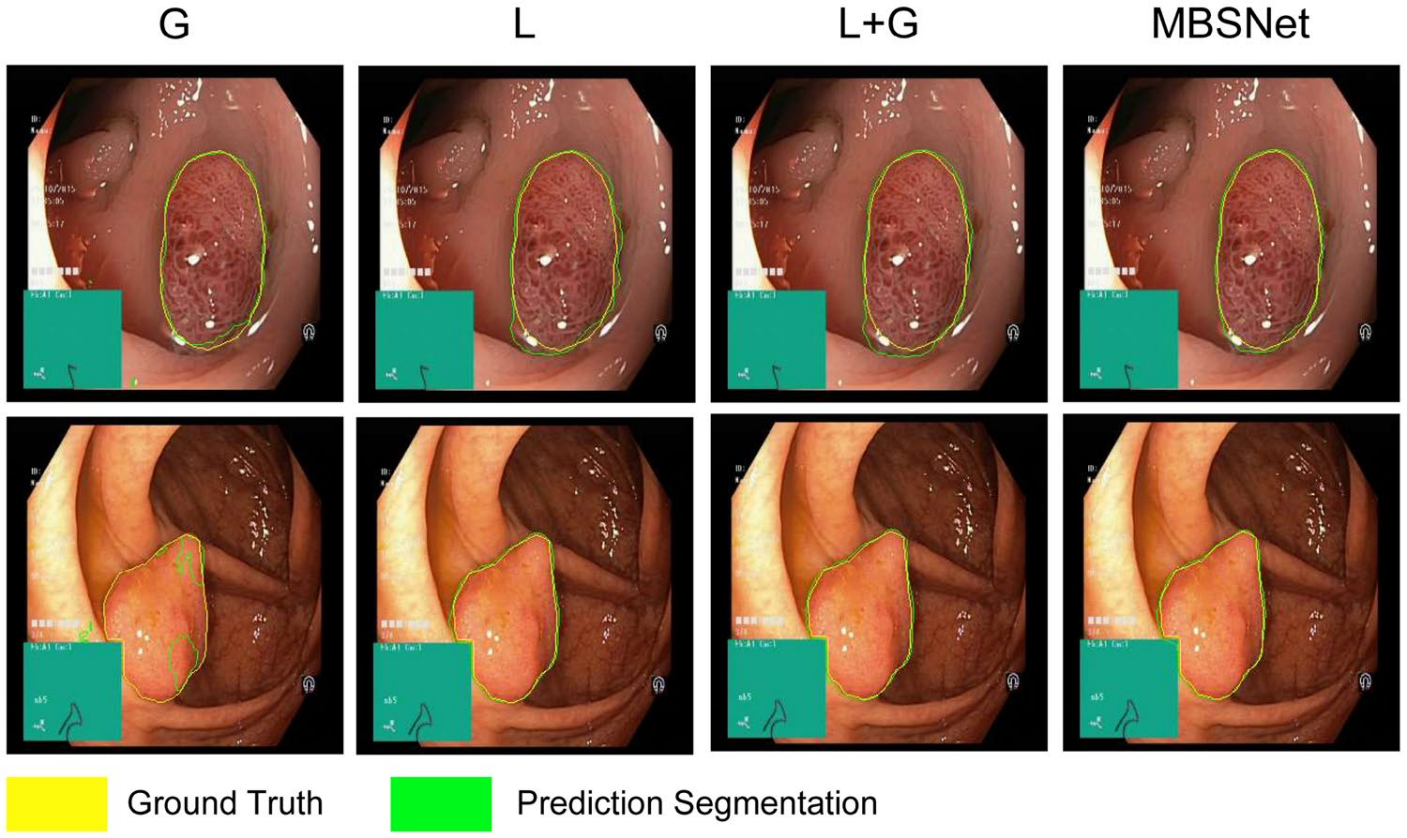
Visual comparison of segmentation by different branches. The level represents the prediction results of different branches of MBSNet for the same image, and the vertical is two representative images from the Kvasir dataset for analysis. The yellow and green lines in the figure represent GT and predicted segmentation results, respectively.

#### Ablation for two-branch training

In this section, we analyze the impact of branches L and G on the results in MBSNet, as well as the performance of branch G and the effectiveness of FCFB in branch F after adding branch L features in the encoder stage. As shown in Table 5, we show the proposed evaluation scores of MBSNet. The experimental results show that each stage is optimized. The full configuration of the MBSNet architecture achieves the best performance.

Figure 8 shows a visual comparison of ablation studies by various branches. The effect of branch L is very close to MBSNet, which shows that local information is very important for the final prediction results in medical segmentation. Then, when branch L and branch G are trained together, the prediction accuracy is slightly reduced. There is no good fusion of local information and global information. Therefore, we propose that FCFB performs information fusion in the decoding stage and obtains higher accuracy.

**Table 5:** Ablation study of our proposed multi-branch, where G represents that only branch G is experimented with, and so is L.

Metrics	G	L	L$+$G	MBSNet
F1	65.54	84.52	81.89	85.29
IOU	53.75	77.12	73.8	77.66

L$+$K refers to the result after removing the FCFB experiment in MBSNet.

## Conclusion

This paper proposes a novel multi-branch medical image segmentation network architecture MBSNet. We believe that fully learning the local and global information in the image can effectively improve the network segmentation performance. Therefore, MBSNet uses the PRM structure to combine the maximum pooling operation and deep convolution to fully learn the complex semantic information in the image. The dilated convolution is used to expand the receptive field of branch G and give the ability to perceive the global information. Based on self-attention, we design a spatial attention module to further enhance global information while reducing complexity and computation. In addition, MBSNet uses a cross-feature fusion method to effectively fuse global information and local information. We validated MBSNet on multiple datasets and showed that the proposed network is lighter and more efficient.

However, although the experimental results of MBSNet on multiple datasets show better performance than UNeXt, the computational complexity is much larger. The network takes up a lot of computation when extracting global information and local information respectively. In addition, the advantages are not obvious on the CT mode dataset and the skin disease dataset. Therefore, further research includes: (1) On the basis of perceiving global and local information, it is necessary to eliminate redundant structures in the network and design lighter modules to enhance feature extraction. (2) For specific medical image segmentation tasks, the segmentation accuracy of MBSNet has to be further improved.

## Data Availability

The datasets generated or analysed during the current study are available in the ISIC Challenge repository, the Simula Datasets repository, the Breast Ultrasound Images Dataset repository, the COVID-QU-Ex Dataset repository, and the Brain MRI segmentation Dataset repository (https://challenge.isic-archive.com/data/#2018, https://datasets.simula.no/kvasir-seg/, https://www.kaggle.com/datasets/aryashah2k/breast-ultrasound-images-dataset, https://www.kaggle.com/datasets/cf77495622971312010dd5934ee91f07ccbcfdea8e2f7778977ea8485c1914df, https://www.kaggle.com/datasets/mateuszbuda/lgg-mri-segmentation).
